# Peculiarities of Resonant Absorption of Electromagnetic Signals in Multilayer Bolometric Sensors

**DOI:** 10.3390/s23031549

**Published:** 2023-01-31

**Authors:** Anna V. Bogatskaya, Nikolay V. Klenov, Alexander M. Popov, Andrey E. Schegolev, Pavel A. Titovets, Maxim V. Tereshonok

**Affiliations:** 1 Department of Physics, Lomonosov Moscow State University, 119991 Moscow, Russia; 2 P. N. Lebedev Physical Institute, Russian Academy of Sciences, 119991 Moscow, Russia; 3 D. V. Skobeltsyn Institute of Nuclear Physics, Lomonosov Moscow State University, 119991 Moscow, Russia; 4 Science and Research Department, Moscow Technical University of Communication and Informatics, 111024 Moscow, Russia

**Keywords:** bolometers, bolometric sensor, semiconductor plasma, absorption of electromagnetic radiation in plasma

## Abstract

We examine the effect of resonant absorption of electromagnetic signals in a silicon semiconductor plasma layer when the dielectric plate is placed behind it both experimentally and numerically. It is shown that such plate acts as a dielectric resonator and can significantly increase the electromagnetic energy absorption in the semiconductor for certain frequencies determined by the dielectric plate parameters. Numerical modelling of the effect is performed under the conditions of conducted experiment. The numerical results are found to be in qualitative agreement with experimental ones. This study confirms the proposed earlier method of increasing the efficiency of bolometric-type detectors of electromagnetic radiation.

## 1. Introduction

The bolometric detection technique is gaining popularity. The basis of this popularity is the ability to “adjust” the spectrum of the electronic ensemble interacting with radiation to certain tasks and frequency ranges [1,2,3,4,5,6,7,8,9,10,11,12]. Various sensors are being developed for a variety of frequency ranges, from gigahertz to infrared. In this article, we consider the features of using well-known resonance effects to solve the problem with the sensitivity of bolometric detectors. We propose to “reverse” the concept of an anti-reflective coating. The parameters of the dielectric substrate under the main working layer with free charges should be selected so that the interference of waves reflected from the boundaries of the resulting heterostructure maximizes the efficiency of their absorption by the electronic ensemble.

The high degree of reflection of metal or doped semiconductor layers, commonly used as sensitive elements in bolometric detectors, leads to a significant reduction in the absorbed energy that decreases the sensitivity of bolometer. A new approach that paves the way to overcome this problem was proposed in [13,14]. It was shown that if one disposes the dielectric plate behind the layer with free charge carriers, the transmitted as well as the absorbed energy of the incident pulse can be dramatically increased for certain frequency bands depending on thickness and permittivity of the dielectric plate. The physics of resonant amplification becomes clearer if one reminds that such a dielectric plate can be treated as an electromagnetic resonator [15]. If the frequency of electromagnetic signal is close to the one of its eigenfrequencies the effective filling of resonator by the electromagnetic wave field becomes possible [15,16,17]. This resonance filling can be accompanied by the resonance absorption of the signal in the plasma layer [13,14].

We would like to mention that the process of the resonant absorption discussed below is significantly different from those proposed in [13]. In [13], the situation when the dielectric resonator is located between the plasma layer with supercritical electron density and perfectly conducting metal surface was considered. In such a situation a closed resonator is formed (the electric field nodes are formed at its boundaries). Moreover, in [13] the plasma layer parameters were chosen to implement the regime of electromagnetic wave tunneling [15,18] which means that the real part of plasma permittivity is negative. It should be noted, such process of resonance optical tunneling is quite similar to the resonance electron transport in heterostructures [19,20,21,22].

Nevertheless, the case of an open resonator is more typical for bolometric applications. Such situation is realized if the electron density in the layer with free charge carriers is below the critical one (it means that the real part of the semiconductor plasma permittivity is positive) and there is no reflective coating on the back side of the structure. In this case the antinodes of the electric wave field appear at the boundaries. From a physical point of view, the appearance of absorption resonances in this case can be simply understood similar to the interference effects in thin films.

In this paper we present the results of both theoretical and experimental investigations of the resonant absorption of gigahertz waves in the structure “conducting layer + dielectric plate”. We employ one or two identical dielectric plates and n-doped silicon semiconducting layer that has considerable absorption in GHz range. Since the real part of permittivity of doped silicon plate appears to be positive for the conditions under study, we have an open resonator. The goal of our study is to demonstrate the resonant enhancement of the absorption in the semiconductor plasma layer if the frequency of incident radiation nearly coincides with one of the resonator’s eigenfrequencies. It will be shown that by adding a second dielectric plate identical to the first one, one can change the resonator eigenfrequencies and, hence, control the position of absorption resonances in the definite frequency range.

## 2. Experimental Setup and Measurements 

**Figure 1:**
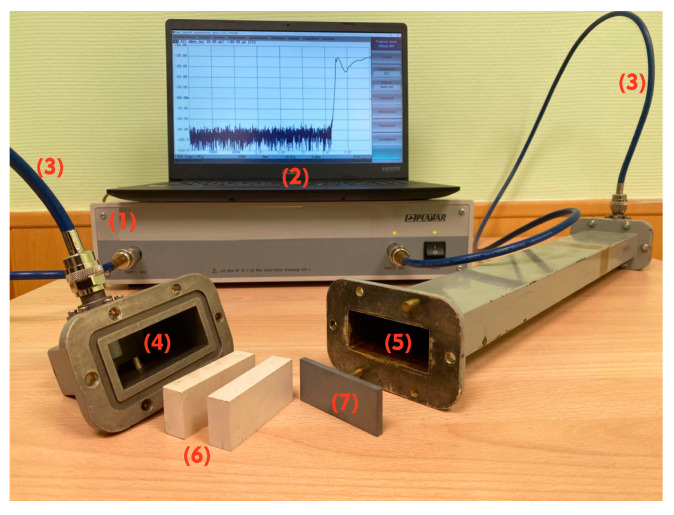
(1) vector circuit analyzer С2409; (2) a laptop; (3) two coaxial cables; (4) two waveguide junctions (CWJ) ADP3B-58x25-NF (internal size 5.8 × 2.5 сm); (5) the waveguide, length 50 сm and internal size 5.8 × 2.5 сm; (6) two dielectric plates, size 5.8 × 2.5 × 1.3 сm, $\varepsilon_{d0}=16.1$ and $tan\;\delta_{\varepsilon }=2\times 10^{-3}$; (7) silicon plate doped with phosphorus of 0.4 cm width with resistivity 47 Ω·cm.

The resonant absorption of electromagnetic signals in multilayer “semiconductor + dielectric” structure was experimentally measured by means of a closed-type waveguide structure. It was a part of our experimental setup displayed in Figure 1. The closed-type waveguide structure, shielded from the environment, consists of a waveguide (length 50 cm, internal size 5.8 × 2.5 сm) and two coaxial waveguide junctions (CWJ). The electromagnetic signal propagating inside the waveguide was detected using an antenna set in the CWJ. Data obtained were processed through the S2409 vector network analyzer (VNA), manufactured by the PLANAR LLC, Chelyabinsk, RUSSIA (https://planarchel.ru/en/company/), which can calculate absorption coefficients of measured signals. All data received from measurements were saved and analyzed on the laptop connected to the vector circuit analyzer. Coaxial cables were pre-calibrated with a VNA prior to measurements to ensure that they did not interfere with the receiving data. The experimental setup was similar to that used in [23].

During the experiment, the propagation of the signal was studied through the following structures: semiconductor layer (silicon doped with phosphorus, resistivity 47 Ω·cm, width 0.4 cm); “semiconductor + dielectric plate” structure (size 5.8 × 2.5 × 1.3 сm with permittivity $\varepsilon_{d0}=16.1$ and loss tangent $tan\;\delta_{\varepsilon }=2\times 10^{-3}$); “semiconductor + double dielectric plate” structure.

The frequency response obtained in the experiment was filtered to remove the influence of the waveguide’s standing waves.

## 3. Results and Discussion

### 3.1. Theoretical Model 

We will start our analysis from the theoretical model of the propagation of the electromagnetic wave in the waveguide in the presence of the “semiconductor + dielectric plate” structure. As the lowest mode H_10_ was used in our experiments we restrict ourselves to the study of only this mode propagation in the rectangular waveguide. This mode is characterized by the transverse electric field, while the magnetic field has also longitudinal components along the direction of propagation [24].

The Helmholtz equation for the electric field strength $E_{\omega }$ in the H_10_ mode in the waveguide has the form [23,24]

(1) $$\frac{\partial^{2}E_{\omega }}{\partial z^{2}}+k_{⊥}^{2}E_{\omega }=0.$$

Here, ω is the angular frequency and $k_{⊥}^{2}=\frac{\omega^{2}}{c^{2}}\left(\varepsilon \left(z\right)-\frac{\omega_{c}^{2}}{\omega^{2}\varepsilon \left(z\right)}\right)$ is the transverse wave number which depends on the permittivity profile along the axis ε(z), c is the speed of light in vacuum. Note that there is the minimal value of radiation frequency that can propagate in the waveguide: in the dielectric-free guide so-called cutoff frequency $\omega_{c}$ is given by the expression $\omega_{c}=\pi с/\ell$, ℓ cm is the internal transverse size of the waveguide. In our experiments it was equal ℓ=5.8 cm. For the given waveguide the single-mode H_10_ propagation regime is realized in the frequency range 2.6–5 GHz [24]. For higher frequencies the multimode regime appears to exist that makes too complicated the interpretation of the experimental data.

**Figure 2:**
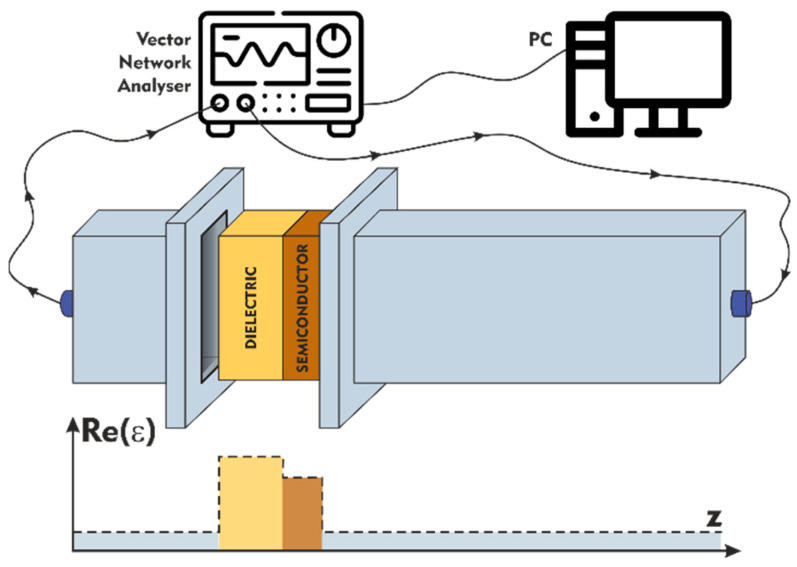
Schematic representation of our experimental setup.

According to the experimental setup [23], the radiation propagates in the waveguide of L=70 cm total length (central waveguide of 50 cm length and two waveguide junctions of 10 cm—see Figure 1). The structure “semiconductor + dielectric” is located at 10 cm from the left end of the waveguide (see Figure 2). For the simulations the origin of reference was chosen at one of the boundaries of the dielectric plate in such a way that ε(z) is given by the expression

(2) $$\varepsilon \left(z\right)=\{\begin{array}{c} \varepsilon_{air},\;-L_{1}\leq z\leq 0,\\ \varepsilon_{d},0<z\leq a,\\ \varepsilon_{p},a<z\leq a+d,\\ \varepsilon_{air},a+d<z\leq L_{0}. \end{array}$$

Here, $L_{1}=10$ cm, $L_{0}=60$ cm, $L_{0}+L_{1}=L$. $\varepsilon_{d}=\varepsilon_{d0}+i\times tan\;\delta_{\varepsilon }$ is the permittivity of the dielectric plate taking into account the losses in material, a and d are the widths of the dielectric plate and silicon semiconductor layer correspondingly. According to Figure 2, the radiation falls on the resonant structure from the right.

In our simulations we put the value a equal to 1.3 cm (single-width dielectric plate) or 2.6 cm (double-width dielectric plate); $\varepsilon_{p}$ is the semiconductor plasma permittivity calculated by the Drude formula [25]:

(3) $$\varepsilon_{p}=\varepsilon_{u}-\frac{\omega_{p}^{2}}{\omega^{2}+\nu^{2}}+i\frac{\omega_{p}^{2}\nu }{\left(\omega^{2}+\nu^{2}\right)\omega }.)$$

Here, $\varepsilon_{u}\approx 12$ is the permittivity of the undoped semiconductor layer, $\omega_{p}^{2}=4\pi e^{2}n_{e}/m^{*}$, ν are the plasma frequency squared and the transport collisional frequency, $n_{e}$ is the density of n-type charge carriers and $m^{*}≅1.08m$ is the electron effective mass in the silicon (*m* is an electron mass)); $\varepsilon_{air}≅1$ is the permittivity of air space in the waveguide. For the semiconductor parameters given in Table 1 one can calculate the electron density in the doped silicon plate as $n_{e}=1.02\times 10^{14}$ cm^−3^ and the value of transport frequency ν as $1.25\times 10^{12}$ s^−1^. As it was mentioned in the introduction, we are dealing with high collisional semiconductor plasma ($\nu >\omega_{p}>\omega$) providing strong absorption in GHz range. Indeed, as $\omega_{p}=5.5\times 10^{11}$ s^−1^ ($f_{p}=\omega_{p}/2\pi =87.3$ GHz), imaginary part of plasma permittivity (Expression (3)) can be estimated as $\omega_{p}^{2}/\nu \omega \sim 10\gg 1$ for $\omega =2.5\times 10^{10}$ s^−1^ (f=ω/2π≈4 GHz).

Numerical integration of the wave Equation (1) with permittivity given by Expressions (2) and (3) was implemented using the software package «Wolfram Mathematica». At the boundary of the waveguide (output signal) we used the Sommerfeld condition:

(4) $$ik_{⊥}E_{\omega }+\frac{\partial }{\partial z}E_{\omega }{|}_{\;z=-L_{1}}=0.$$

It means that we neglect the effect of re-reflection at the ends of the waveguide. Our numerical procedure assumes normalization of the obtained solution to the given incoming radiation flux («shooting» method). To calculate the fraction of absorbed radiation flux $\Phi_{a}^{}$ we use the following expression:

(5) $$\Phi_{a}=1-\Phi_{t}-\Phi_{r},$$

where $\Phi_{t}$ and $\Phi_{r}$ are the transmitted and reflected radiation fluxes correspondingly normalized to the incident radiation flux.

### 3.2. Results and Discussion

Usually, the radiation weakly affects the free carriers’ ensemble in the semiconductor, which leads to a limited efficiency of the bolometer. The main idea of our study consists in the effect of enhancing of the fraction of absorbed radiation energy at definite frequency bands when the dielectric plate is placed behind the absorbing semiconductor layer. The maximum absorption can be achieved for frequencies similar to those for standing waves in the dielectric layer (eigenfrequencies). If the radiation frequency matches the eigenfrequencies, the signal energy concentrates at the junction of the dielectric and the semiconductor and can be detected there more efficiently. We show how the frequencies with maximum absorption depend on the dielectric parameters. It is important to mention that in contradiction with [11,12,13,14,15] where the metal plasma acted as tunnel barrier for electromagnetic radiation here the semiconductor layer can be considered as a resonator but with low Q-factor (high absorption) since in our conditions the real part of permittivity (3) appears to be more than unity. Thus, the structure represents a system of two coupled resonators.

**Figure 3:**
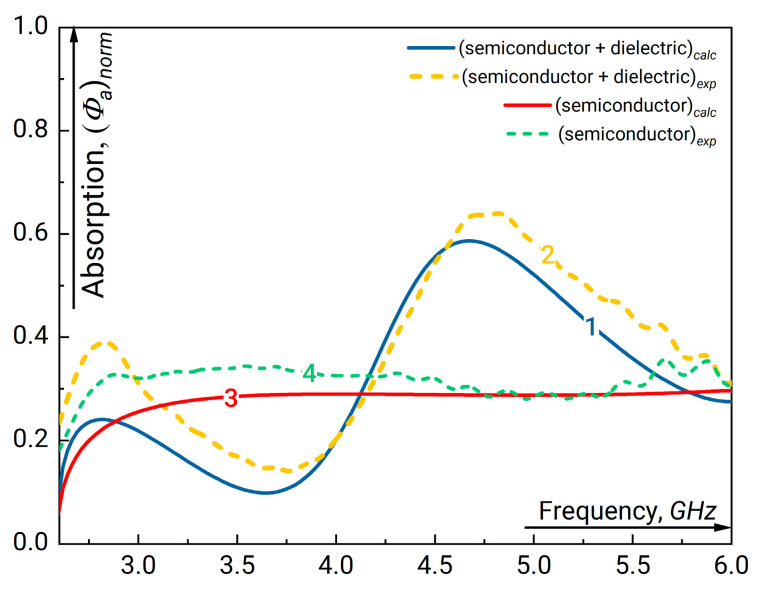
The fraction of absorbed GHz radiation normalized to the incoming radiation flux measured at the output of waveguide in the presence of resonant structure “semiconductor + single dielectric” (1, 2) and semiconductor layer only (3, 4) versus the linear signal frequency. The parameters of the undoped dielectric plate are $\varepsilon_{d}=16.1$, width a=1.3 cm. For the semiconductor plate d=0.4 cm and ρ=47 Ω·cm (see Table 1). “1” and “3” correspond to the simulation results “2” and “4” are the experimental curves.

**Figure 4:**
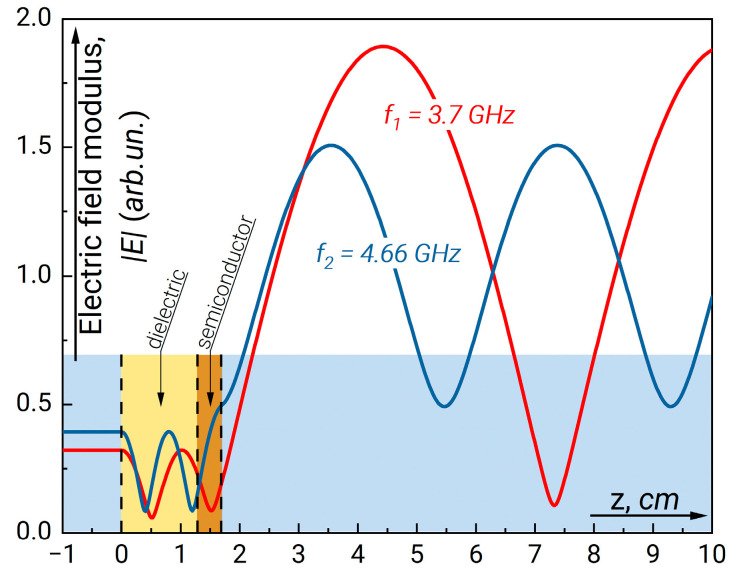
The field modulus distribution along the waveguide for *f*_1_ = 3.7 GHz (1) (minimum absorption) and *f*_2_ = 4.66 GHz (maximum absorption). The parameters of resonance structure correspond to those in Figure 3.

We will start our analysis from the structure with a thin semiconductor and a single-width dielectric plate. The results on GHz radiation absorption in semiconductor plate with and without the dielectric plate behind calculated using the Expression (5) compared with experimental data are presented in Figure 3. Here, we assume that the absorption in dielectric plate characterized by the loss tangent $tg\;\delta_{\varepsilon }$ is negligible. First, one can see considerable increase of absorption near the frequency 4.7 GHz. This frequency approximately corresponds to the condition when an integer number of wavelengths fits the length of the considered structure. On the contrary, the absorption minimum corresponds to the situation when an odd number of wave quarters fits along the resonator length. The distributions of the field absolute value inside and outside the resonance structure for the frequencies corresponding to minimum and maximum absorption are plotted in Figure 4. Data in Figure 4 demonstrates that high absorption occurs when the wave field effectively fills the region of semiconductor layer. As can be seen, there is an antinode of the field at both ends of the resonator structure.

**Figure 5:**
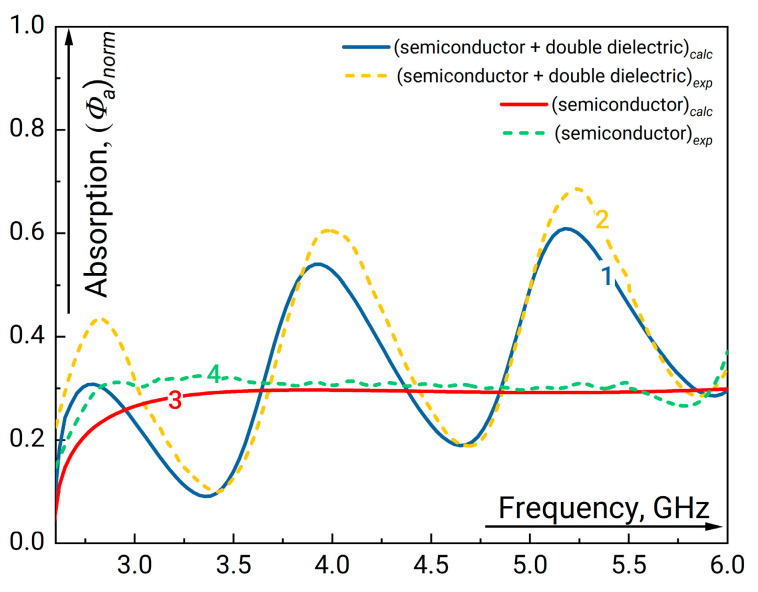
The fraction of absorbed GHz radiation normalized to the incoming radiation flux measured at the output of waveguide in the presence of resonant structure “semiconductor + double dielectric” (1, 2) and semiconductor layer only (3, 4) versus the linear signal frequency. The parameters of undoped double dielectric plate are $\varepsilon_{d}=16.1$, width a=2.6 cm. The parameters of semiconductor plate with d=0.4 cm and ρ=47 Ω·cm (see Table 1). Here, “1” and “2” are the experimental curves, “3” and “4” correspond to the simulation results.

**Figure 6:**
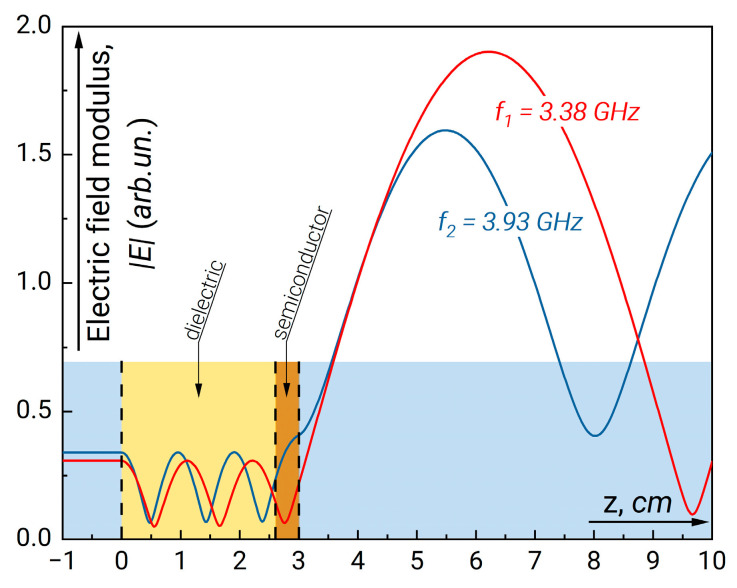
The field modulus distribution along the waveguide for *f*_1_ = 3.38 GHz (1) (minimum absorption) and *f*_2_ = 3.93 GHz (maximum absorption). The parameters of resonance structure correspond to those in Figure 5.

By changing the thickness and permittivity of the dielectric plate, one can control the position of the absorption maxima. Figure 5 shows the position of maxima when the double-width dielectric plate is placed behind the semiconductor layer. In this case, the absorption resonances are located close to each other while the width of each resonance narrows. The field distribution for the structure with double dielectric plate for the frequencies at central (second) maximum and the adjacent minimum is depicted in Figure 6.

Finally, we should stress that the recent idea that a resonant increase in the transmission coefficient leads to an increase in the fraction of absorbed energy turns out to be valid both in the regime of resonant tunneling of signal through the plasma layer (when the real part of the plasma permittivity is negative) and in the case where the plasma serves as a resonator with high absorption. However, the relative position of the transmission and absorption resonances will strongly depend on plasma parameters and the specific regime that turns out to be implemented within the framework of a concrete problem. It is also important to mention that in situation under the study (the thickness of the silicon layer 0.4 cm, thermal conductivity of the silicon ~$8.3\times 10^{-3}$ cm^2^/s) the thermal flux at the backward size of the semiconductor layer will appear on times of several seconds. Hence, for smaller pulse durations, there is no necessity to consider the thermal contact between the conducting layer and the dielectric plate behind it. If the conducting layer is thin enough, such a problem may be of importance and the thermal flux from the absorbing layer to the dielectric substrate should be taken into account. To avoid this problem, the special vacuum gap between both plates can be organized (We do not discuss this situation in detail as it requires the analysis for definite structure parameters that can significantly differ from those in our study).

## 4. Conclusions

To conclude, we have demonstrated experimentally and numerically the effect of resonant absorption of GHz signal in semiconductor layer in the presence of dielectric plate behind it. The experimental data were found to be in a good agreement with the theoretical calculations. This fact proves the ability to increase the bolometric sensors efficiency significantly in quite an easy way. The results of the study are urgent for the development of a recently proposed method for increasing the sensitivity of bolometric detection, which is based on the embedding of a dielectric substrate behind the sensitive element of the detector. Despite the fact that the experimental results demonstrate the effect in the gigahertz range, the corresponding method can be adapted to devices operating in THz and IR ranges.

## Figures and Tables

**Table 1:** Silicon plate parameters.

	Measured Electrical Resistivity, ρ, Ω·cm	Width, *d*, cm	Electron Mobility in Silicon at a Room Temperature
n-doped silicon plate	47 ± 3	0.4	μ=1300 cm^2^/(V∙s)

## Data Availability

Not applicable.
